# Managing Antithrombotic Treatment in Intracerebral Hemorrhage and Ischemic Stroke Due to Cerebral Amyloid Angiopathy With Supporting Pathology

**DOI:** 10.7759/cureus.111206

**Published:** 2026-06-20

**Authors:** Chelsea Mae N Nobleza, Charlene Marie P Bustamante, Ibet Marie Y Sih, Raymond L Rosales

**Affiliations:** 1 Neurology, St. Luke's Medical Center, Quezon City, PHL; 2 Neurological Surgery, St. Luke's Medical Center, Quezon City, PHL; 3 Neurological Surgery, University of the Philippines-Philippine General Hospital, Manila, PHL; 4 Neurology, Movement Disorders, and Neurophysiology, University of Santo Tomas Hospital, Manila, PHL; 5 Neurology, Movement Disorders, and Neurophysiology, St. Luke's Medical Center, Quezon City, PHL

**Keywords:** cerebral amyloid angiopathy, cilostazol, ischemic stroke, modified boston criteria, stroke workup

## Abstract

Cerebral amyloid angiopathy (CAA) is a small vessel disease marked by β-amyloid deposition within cortical and leptomeningeal vessels. It is associated with a range of hemorrhagic manifestations, including lobar intracerebral hemorrhage (ICH), cortical superficial siderosis, and convexity subarachnoid hemorrhage (cSAH). In addition to hemorrhagic complications, cerebral ischemic events may occur due to impaired vascular reactivity and reduced cerebral perfusion. The coexistence of ischemic and hemorrhagic strokes in patients with biopsy-confirmed CAA poses significant diagnostic and therapeutic challenges, particularly in relation to antithrombotic use. A 74-year-old woman with type 2 diabetes mellitus and dyslipidemia initially presented with transient focal neurological deficits and subsequently developed left parietal cSAH along with multiple acute infarcts involving the left parietal, temporal, and centrum semiovale regions, for which aspirin and rivaroxaban were initiated. Over the following years, she developed progressive cognitive decline. Three years after her ischemic event, she experienced a lobar ICH in the right frontotemporoparietal region and later suffered another ischemic stroke, prompting treatment with cilostazol. This case illustrates the coexistence of ischemic and hemorrhagic pathology in CAA. Recurrent lobar ICH is a characteristic feature of CAA; however, ischemic events may also occur during its course and can adversely affect prognosis. The lack of clear guidance regarding antithrombotic therapy further complicates management. In this context, cilostazol was selected due to its favorable safety profile in patients at high risk of hemorrhage. This approach underscores the importance of individualized treatment strategies in CAA.

## Introduction

Cerebral amyloid angiopathy (CAA) is characterized by β-amyloid protein deposition in cortical and leptomeningeal blood vessel walls and often presents with cerebral hemorrhage [1]. CAA is categorized into four groups based on the Boston Criteria: definite CAA, probable CAA with supporting pathology, probable CAA, and possible CAA [2]. The recent update to the Boston Criteria (version 2.0) has incorporated emerging MRI markers of CAA, which are divided into characteristic hemorrhagic MRI biomarkers, including lobar cerebral microbleeds, lobar intracerebral hemorrhages (ICHs), cortical superficial siderosis (cSS), and convexity subarachnoid hemorrhage (cSAH), as well as nonhemorrhagic white matter markers, including visible perivascular spaces in the centrum semiovale and the white matter hyperintensity multispot pattern [3].

Although CAA most commonly presents with ICH, the co-occurrence of hemorrhagic and ischemic lesions has been observed in CAA in the absence of cardiovascular risk factors [4]. Impaired vasoreactivity in CAA causes ischemic damage due to a mismatch between perfusion and metabolic demand [4]. White matter hyperintensities of presumed vascular origin, predominantly in the posterior cortical areas, have been shown to be more severe in patients with CAA. Pathologic examination of these hyperintensities has demonstrated an ischemic origin, such that increased white matter hyperintensity volume is associated with lower cerebral perfusion [4].

The occurrence of both ischemic stroke and ICH in CAA presents management challenges. To date, no therapeutic consensus regarding anticoagulant or antithrombotic use following ischemic stroke in patients with CAA has been established. The risk of ischemic stroke without antithrombotic therapy must be weighed carefully against the risk of ICH associated with antithrombotic therapy [5]. In this paper, we report the case of a 74-year-old with CAA who presented with lobar ICH and a subsequent ischemic stroke.

This article was previously presented in part as a poster presentation at the 2026 American Academy of Neurology (AAN) Annual Meeting, held on April 18-22, 2026, in Chicago, IL, USA.

## Case presentation

A 74-year-old female with known type 2 diabetes mellitus and dyslipidemia presented three years earlier with recurrent episodes of slurred speech and right-sided facial droop lasting one to two minutes, for which no immediate imaging was performed. In the same year, she developed a left parietal cSAH (Figure 1).

**Figure 1:**
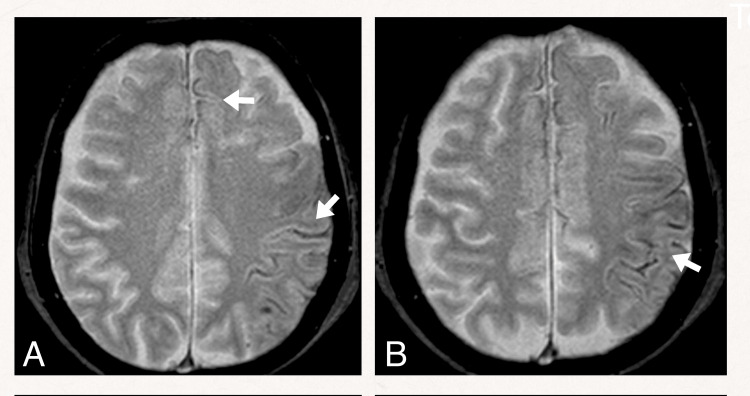
GRE MRI sequence. (A) GRE susceptibilities in the left frontal area, as denoted by the white arrows. (B) GRE susceptibilities in the left frontoparietal area, as denoted by the white arrow. GRE, gradient-recalled echo

One month after her diagnosis of cSAH, she presented with recurrent slurred speech and had a National Institutes of Health Stroke Scale (NIHSS) score of 2, with a shallow right nasolabial fold and mild dysarthria, which were transient focal neurologic episodes (TFNEs) [6]. She was started on aspirin 100 mg once daily and rivaroxaban 10 mg once daily for an unrecalled indication. In the interim, she developed increasing forgetfulness, repeatedly asking questions and getting lost in familiar places while remaining fluent and able to comprehend. She was able to carry out activities of daily living and instrumental activities of daily living but was no longer able to handle money or manage her business. She was started on rivastigmine 4.6 mg patch once daily, with noted improvement in cognitive impairment.

In May 2025, the patient presented with left-sided weakness, slurred speech, and left-sided facial droop without headache or vomiting. Neurologic examination revealed lid apraxia, left homonymous hemianopsia by threat, left central facial palsy, severe dysarthria, left-sided hemiplegia, and a positive Babinski sign on the left. MRI performed during the Brain Attack Code demonstrated a right frontotemporoparietal hemorrhage with subfalcine herniation and intraventricular extension (Figure 2).

**Figure 2:**
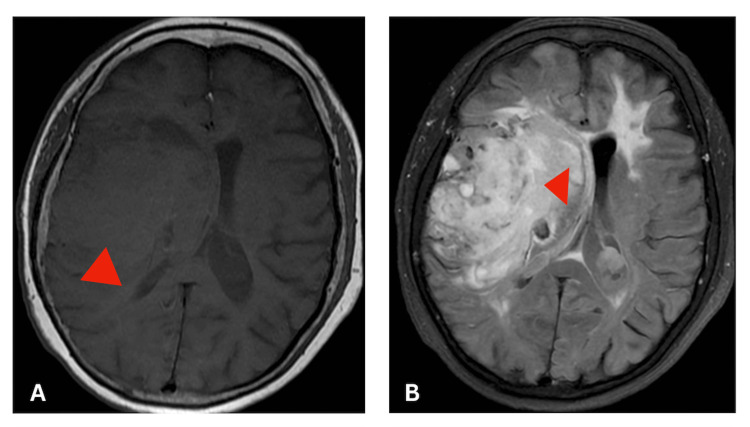
MRI T1- and T2-weighted FLAIR sequences showing an acute lobar ICH. (A) T1 isointense intraparenchymal hemorrhage in the frontal area causing effacement of the right lateral ventricle, as denoted by the red arrowhead. (B) T2 hyperintense intraparenchymal hemorrhage causing effacement of the right lateral ventricle, as denoted by the red arrowhead. ICH, intracerebral hemorrhage

The patient underwent right frontal craniotomy with hematoma evacuation, and tissue biopsy revealed CAA (Figure 3).

**Figure 3:**
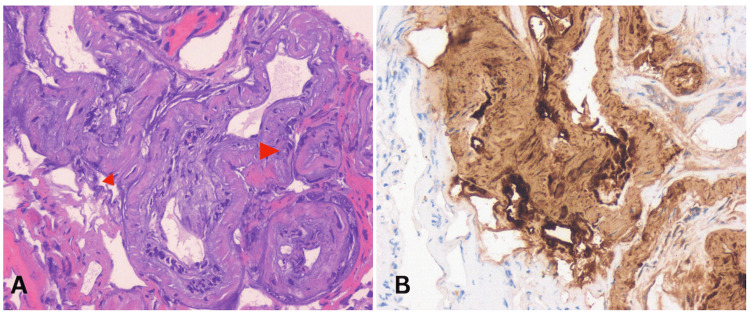
(A) Cerebral blood vessels with acellular, homogeneous, eosinophilic material in their walls, as denoted by the arrowheads. (B) Cerebral blood vessels staining positive for β-amyloid.

During the same admission, the patient developed an acute infarct in the left parieto-occipital lobe (Figure 4A).

**Figure 4:**
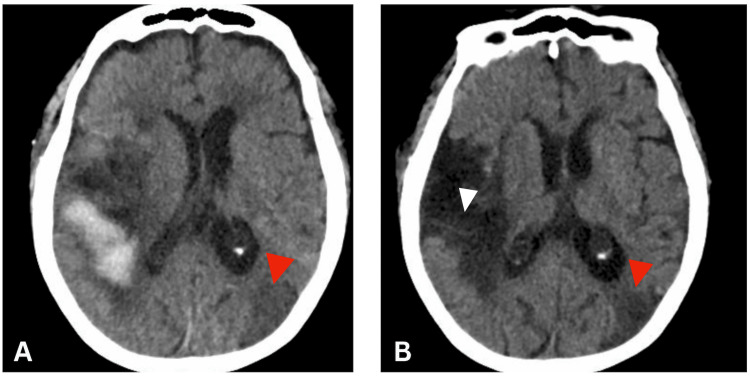
(A) Plain cranial CT scan revealing a new hypodensity in the left parieto-occipital lobe, as denoted by the arrowhead. (B) Repeat plain cranial CT scan obtained 28 days after the ischemic stroke showing resolution of the ICH in the right parietotemporal region, with no new hemorrhages, as denoted by the white arrowhead, and no hemorrhagic conversion of the left parieto-occipital infarct, as denoted by the red arrowhead.

On day 2 post-ictus, the patient was started on cilostazol 50 mg twice daily, which was eventually increased to 100 mg twice daily at discharge. A repeat CT scan obtained 28 days after initiation of cilostazol showed no new hemorrhages or hemorrhagic conversion of the previously noted acute infarct in the left parieto-occipital lobe (Figure 4B). Seven days after discharge, she developed a preferential gaze to the right with versive head turning to the right lasting less than five minutes. Levetiracetam 500 mg twice daily was started for seizure prophylaxis and was increased to 1 g twice daily, with no seizure recurrence. At follow-up three months after the ICH event, the patient was doing well, with no new neurologic symptoms or seizures. She had started physical therapy twice weekly.

## Discussion

Probable CAA with supporting pathology requires a clinical history of spontaneous ICH, TFNEs, cSAH, or cognitive impairment or dementia, along with a tissue diagnosis from an evacuated hematoma or cortical biopsy in the absence of another diagnostic lesion [3]. The patient in this report satisfied the Boston Criteria for probable CAA with supporting pathology based on a positive cortical biopsy for β-amyloid and the clinical findings of lobar hemorrhage, cSAH, cSS, and cognitive impairment.

This patient also had an ischemic stroke in the left parieto-occipital area during the same admission. A similar case was reported in 2019 in which the patient presented with simultaneous ICH and ischemic stroke [7]. Although the association of cSAH and ICH is strongest in CAA, the absolute risk is similar for both ischemic stroke and ICH after the diagnosis of CAA [8]. In patients with cSS, an increased frequency of both ischemic and hemorrhagic strokes has been observed [9]. Similarly, in patients with cSAH due to CAA, the absolute event rate was 5.1% per patient-year, and the median time to ischemic stroke was 1.3 years [9]. Amyloid deposition in blood vessels is thought to cause endothelial dysfunction and vessel wall thickening, leading to hypoperfusion. Furthermore, patients with CAA have been shown to have reduced mean blood flow velocity, which correlates with greater white matter hyperintensity volume [10].

Initiating antithrombotic medications in patients with CAA who have experienced both ICH and ischemic stroke presents management challenges. In patients with cSS, single antiplatelet or anticoagulant therapy was not associated with an increased risk of cerebrovascular events [9]. An observational study found that aspirin lowered the risk of ischemic stroke without increasing the rate of ICH [11]. The REstart or STop Antithrombotics Randomized Trial (RESTART) showed that restarting antiplatelet therapy appeared to reduce ICH, although this study included only a few patients with probable CAA [12,13]. Our patient was started on cilostazol. A subgroup analysis of the Prevention of Cardiovascular Events in Asian Ischemic Stroke Patients With High Risk of Cerebral Hemorrhage (PICASSO) trial, which compared aspirin and cilostazol in patients with ischemic stroke and a high risk of cerebral hemorrhage, found that hemorrhagic stroke was less frequent in the cilostazol group than in the aspirin group among patients with multiple microbleeds (1 versus 13 events; hazard ratio, 0.08 (95% CI: 0.01-0.61); P = 0.01). In addition, cilostazol reduced the rate of any stroke in patients with mild-to-moderate white matter hyperintensities [14]. Evidence has also shown that cilostazol has protective effects against cognitive decline in patients with Alzheimer’s disease [15].

## Conclusions

CAA significantly contributes to lobar ICH; however, it can also present with ischemic stroke, and the occurrence of both ischemic and hemorrhagic events in our patient highlights the diverse clinical spectrum of CAA. The management of CAA presents significant challenges. The risk of recurrent hemorrhage must be carefully weighed against the potential benefits of antithrombotic therapy in mitigating ischemic events. The administration of cilostazol in this case provided a pragmatic therapeutic option for patients with CAA who are at high risk of hemorrhage. This case highlights the importance of individualized treatment approaches, vigilant monitoring, and further studies to guide evidence-based management of patients with CAA manifesting both ischemic and hemorrhagic strokes.
